# Supplementary material for: shRNAs Targeting a Common KCNQ1 Variant Could Alleviate Long-QT1 Disease Severity by Inhibiting a Mutant Allele
Source: Int J Mol Sci. 2022 Apr 6;23(7):4053. doi: 10.3390/ijms23074053 (PMC9000197; doi:10.3390/ijms23074053)
Supplement: Supplementary file 1 [file ijms-23-04053-s001.zip › ijms-1639066-supplementary.pdf]

Lucía Cócera-Ortega et al. shRNAs Targeting a Common *KCNQ1* Variant Could Alleviate Long-QT1 Disease Severity by Inhibiting a Mutant Allele. *Int. J. Mol. Sci.* 2022

Supplemental tables and figures

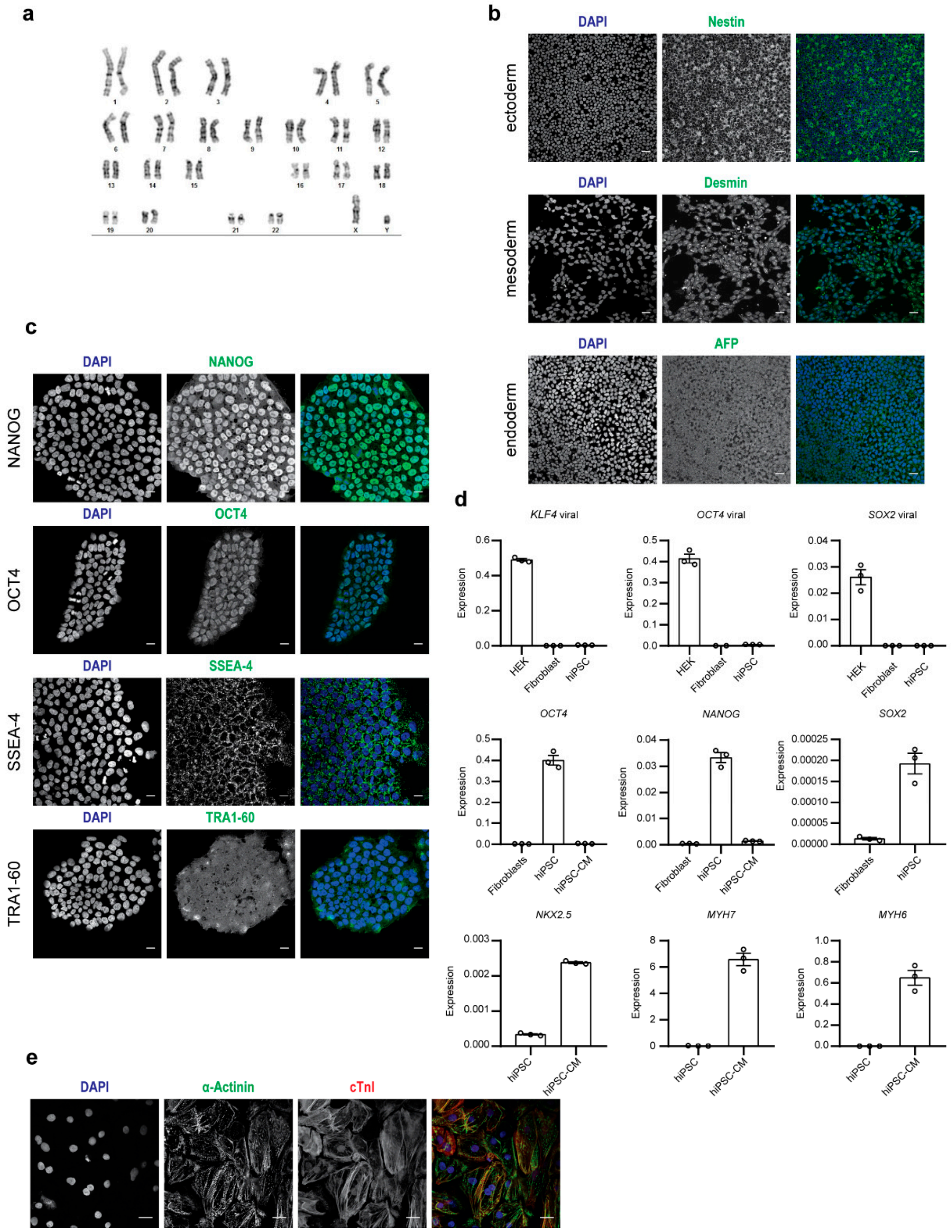

Figure S1. Characterization of Line 2. a) Karyotype analysis of hiPSCs of Line 2. **b)** Immunofluorescence after in vitro 3 lineage differentiation. Scale bars 20 µm. **c)** Immunofluorescence of pluripotency markers in undifferentiated hiPSCs. Scale bars 20 µm. **d)** Downregulation of viral genes in hiPSCs after induction of pluripotency with HEK cells transfected with the viral plasmids as positive control, expression of pluripotency markers OCT4, NANOG and SOX2 in undifferentiated hiPSCs and expression of cardiomyocyte markers NKX2.5, MYH7 and MYH6 in hiPSC-CMs measured by RT-qPCR. Error bars indicate SEM. **e)** Immunofluorescence of cardiomyocyte markers in hiPSC-CMs. Scale bars 20 µm. For all immunofluorescence pictures the separate panels

on the left are merged into the rightmost panel with colour coding as indicated above the separate panels. Characterization of line 1 is included elsewhere (Tijsen et al. manuscript in draft).

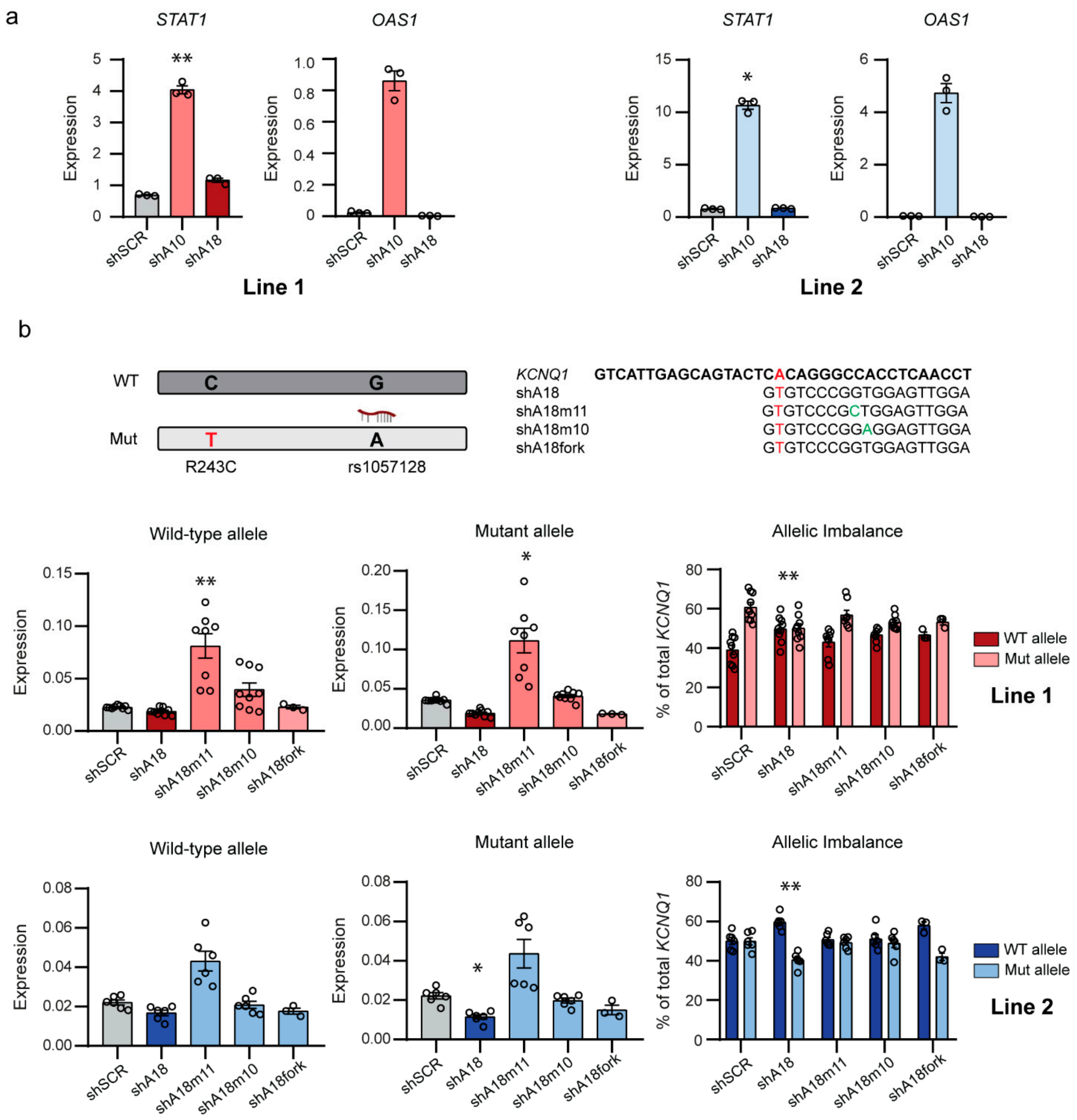

Figure S2. Introduction of additional mismatches does not improve allele-specificity of shRNAs targeting the A allele of SNP rs1057128 on the mutant *KCNQ1* allele. a) Relative mRNA expression of interferon response genes *STAT1* and *OAS1* for the most efficient shRNAs targeting the A allele of rs1057128 in hiPSC-CMs of Line 1 (red; left) and Line 2 (blue; right). **b)** Top, schematic representation of the shRNAs targeting the A allele of rs1057128 on the mutant *KCNQ1* allele with the mismatch positions indicated (original mismatch in red and additional mismatches in green). Middle and bottom, allele-specific relative mRNA expression of the wild-type and mutant *KCNQ1* alleles

(left two panels) and the allelic expression of the wild-type and mutant *KCNQ1* alleles presented as % of total *KCNQ1* expression (right panel). In red hiPSC-CMs of Line 1 (middle, n=3-9) and in blue hiPSC-CMs of Line 2 (bottom, n=3-6). **P*<0.05; ***P*<0.025compared to shSCR negative control; error bars indicate SEM.

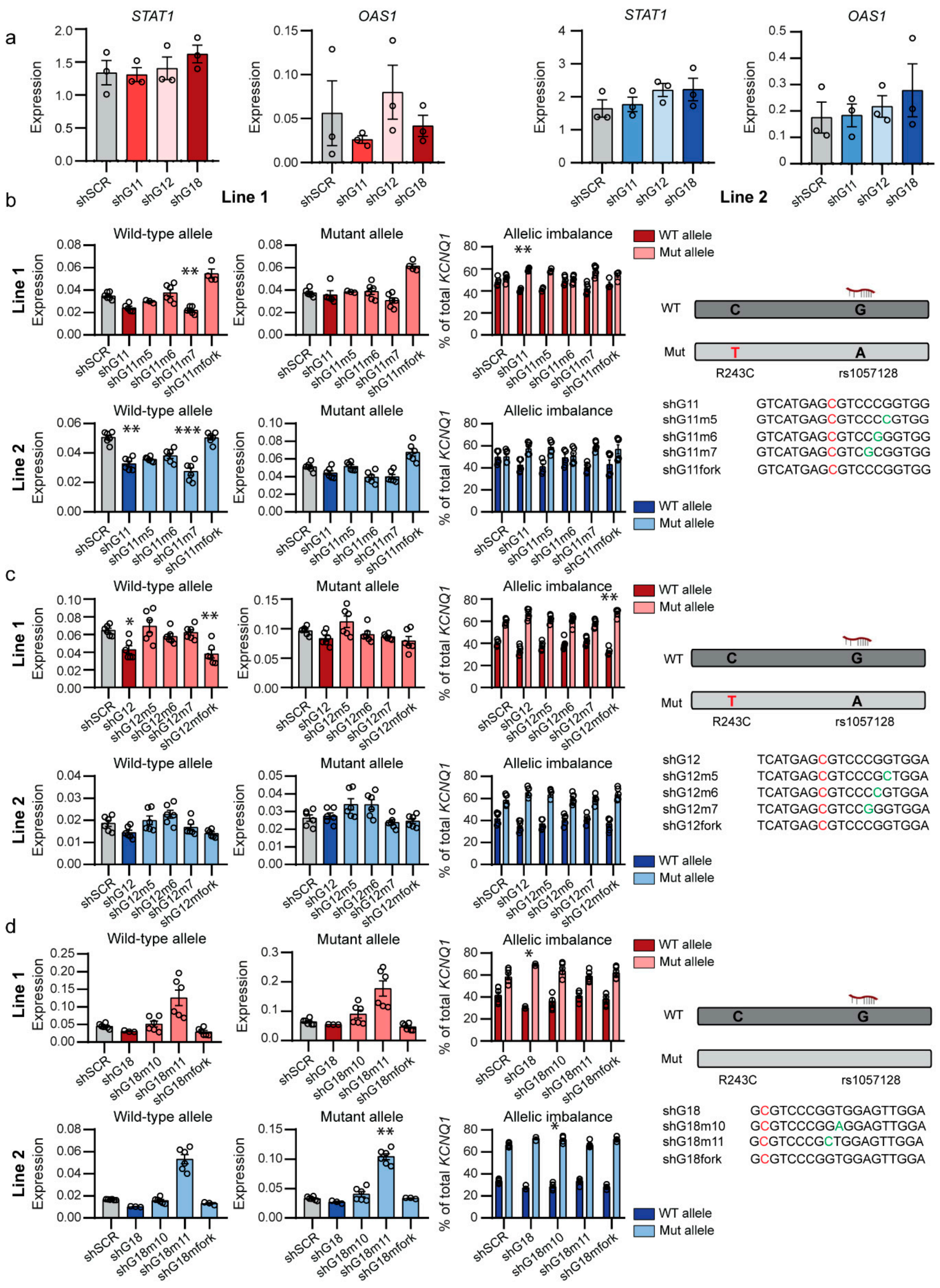
a
STAT1
OAS1
STAT1
OAS1
Expression
shSCR
shG11
shG12
shG18
Line 1
Line 2
b
Wild-type allele
Mutant allele
Allelic imbalance
Line 1
Line 2
Expression
% of total KCNQ1
shSCR
shG11
shG11m5
shG11m6
shG11m7
shG11mfork
WT allele
Mut allele
WT
Mut
C
G
T
A
R243C
rs1057128
shG11 GTCATGAGCGTCCCGGTGG
shG11m5 GTCATGAGCGTCCCCGTGG
shG11m6 GTCATGAGCGTCCGGGTGG
shG11m7 GTCATGAGCGTCGCGGTGG
shG11fork GTCATGAGCGTCCCGGTGG
c
shG12
shG12m5
shG12m6
shG12m7
shG12mfork
shG12 TCATGAGCGTCCCGGTGGA
shG12m5 TCATGAGCGTCCCGCTGGA
shG12m6 TCATGAGCGTCCCCGTGGA
shG12m7 TCATGAGCGTCCGGGTGGA
shG12fork TCATGAGCGTCCCGGTGGA
d
shG18
shG18m10
shG18m11
shG18mfork
shG18 GCGTCCCGGTGGAGTTGGA
shG18m10 GCGTCCCGGAGGAGTTGGA
shG18m11 GCGTCCCGCTGGAGTTGGA
shG18fork GCGTCCCGGTGGAGTTGGA

Figure S3. Introduction of additional mismatches does not improve allele-specificity of shRNAs targeting the G allele of SNP rs1057128 on the wild-type *KCNQ1* allele. a) Relative mRNA expression of interferon response genes *STAT1* and *OAS1* for the most efficient shRNAs targeting the G allele of rs1057128 in hiPSC-CMs of Line 1 (red; left) and Line 2 (blue; right). **b-d)** Right, schematic representation of the shRNAs targeting the G allele of rs1057128 on the wild-type *KCNQ1* allele with the mismatch positions indicated (original mismatch in red and additional mismatches in green). Left, allele-specific relative mRNA expression of the wild-type and mutant *KCNQ1* alleles and the allelic expression of the wild-type and mutant *KCNQ1* alleles presented as % of total *KCNQ1* expression. In red hiPSC-CMs of Line 1 (top; n=3-9) and in blue hiPSC-CMs of Line 2 (bottom; n=3-6). **P*<0.05; ***P*<0.025; ****P*<0.001 compared to shSCR negative control; error bars indicate SEM.

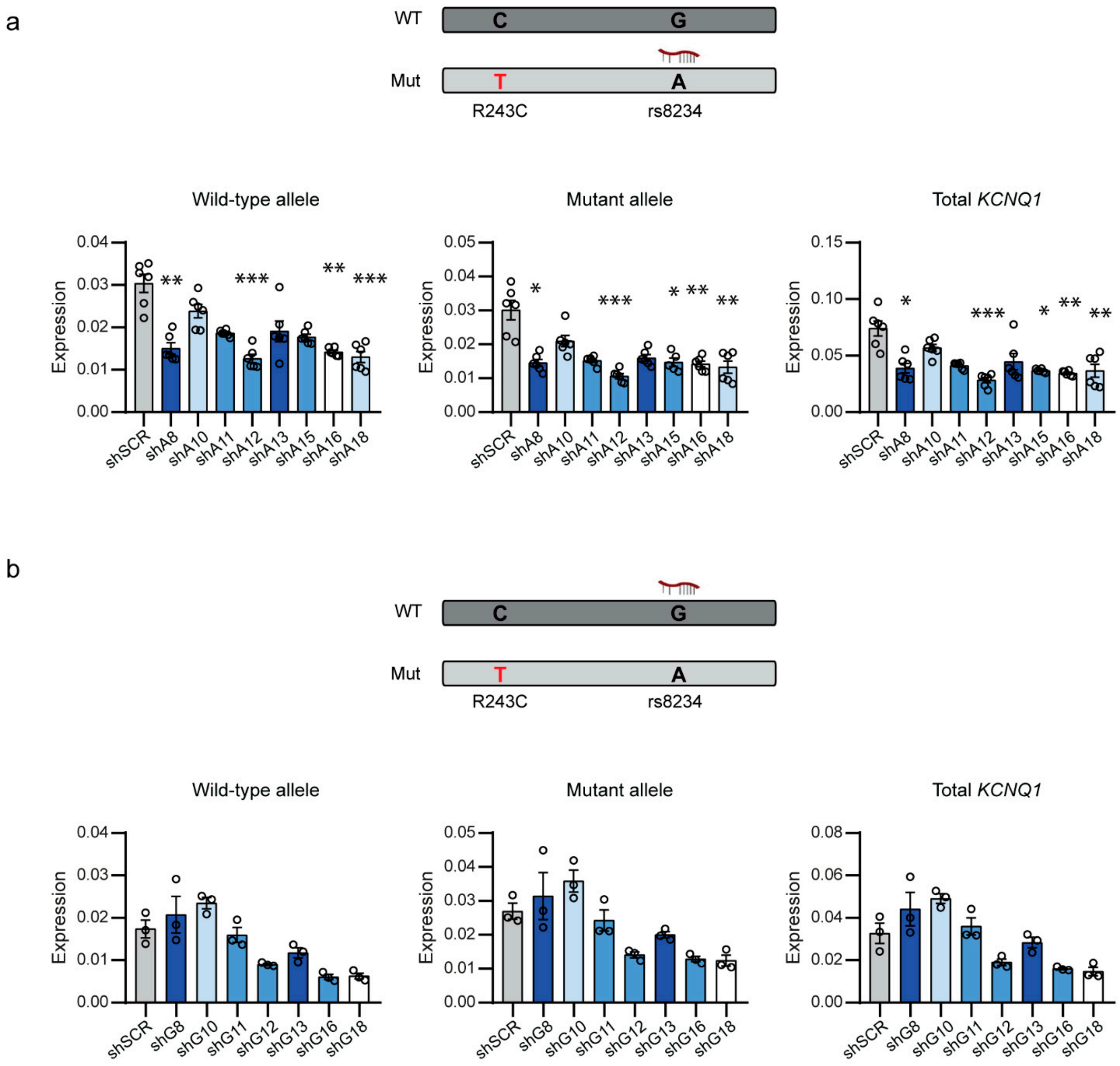

Figure S4. Allele specific shRNAs targeting SNP rs8234 in the 3'UTR of *KCNQ1*. a) Top, representation of the targeting of the A allele of rs8234 on the mutant *KCNQ1* allele in hiPSC-CMs of Line 2. Bottom, relative allele-specific expression of the wild-type and mutant *KCNQ1* allele and total *KCNQ1* (n=6). **b)** Top, representation of the targeting of the G allele of rs8234 on the wild-type *KCNQ1* allele. Bottom, relative allele-specific expression of the wild-type and mutant *KCNQ1* allele and total *KCNQ1* measured by qRT-PCR (n=3). **P*<0.05; ***P*<0.025; ****P*<0.001 compared to shSCR negative control; error bars indicate SEM.

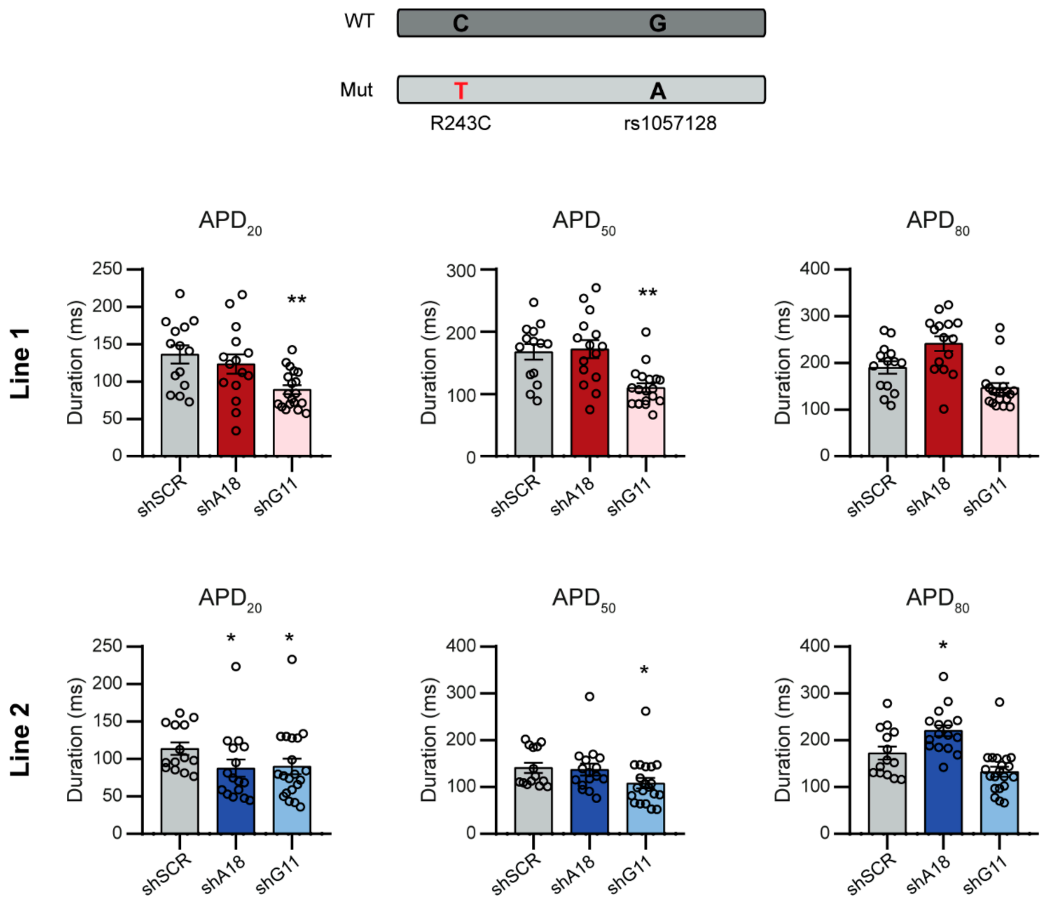

Figure S5. Action potential duration is affected by shifts in allelic balance. Schematic representation of the SNP and mutation in *KCNQ1* (top) and action potential duration at 20, 50 or 80% of repolarization (APD_{20}, APD_{50}, and APD_{80}, respectively) of optical action potentials derived from ArcLight fluorescence changes in hiPSC-CMs from Line 1 (red) or Line 2 (blue) treated with either negative control shSCR, shA18 targeting the mutant *KCNQ1* allele or shG11 targeting the wild-type *KCNQ1* allele stimulated at 2 Hz. *P<0.05; **P<0.025compared to shSCR negative control; error bars indicate SEM.

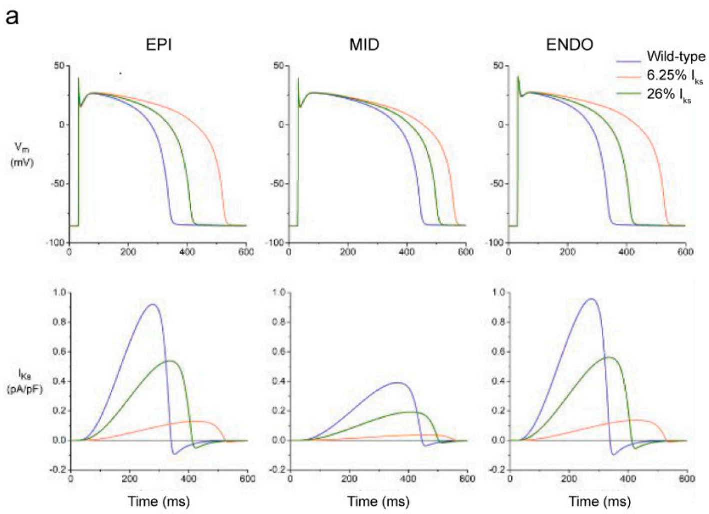

b

	APD$_{90}$ (ms)		
	**EPI**	**MID**	**ENDO**
**Wild-type**	311	417	309
**6.25% I_{ks}**	495	531	501
**26% I_{ks}**	382	473	383

Figure S6. Effects of changes in the slow delayed rectifier potassium current (I_{Ks}) in a human ventricular cell model. a) Membrane potential (V_m; top) and associated I_{Ks} (bottom) at 1 Hz stimulation under wild-type conditions, upon reduction of functional K_v7.1 channels to 1/16th as expected when both alleles are equally expressed (6.25%) or to 26% as expected when the mutant *KCNQ1* allele is downregulated by 60%. **b)** Associated APD_{90} values in the epicardial (EPI), midmyocardial (MID) and endocardial (ENDO) versions of the human cardiomyocyte model.

Table S1

Antibody	Supplier	Species	Dilution	Cat. number
**OCT-4**	STEMCELL Technologies Vancuver, Canada	Mouse	1:200	60093
**SSEA-4**	STEMCELL Technologies Vancuver, Canada	Mouse	1:100	60062
**Tra1-60**	STEMCELL Technologies Vancuver, Canada	Mouse	1:100	60064
**NANOG**	Peprotech Rocky Hill, NJ,USA	Rabbit	1:200	500-P236
**α-Actinin**	Abcam Cambridge, UK	Rabbit	1:500	68167
**cTnI**	HyTest Turku, Finland	Goat	1:400	4T21/2

**Nestin**	STEMCELL Technologies Vancuver, Canada	Mouse	1:1000	60091
**AFP**	ThermoFisher Rockford, IL, USA	Rabbit	1:100	PA5-16658
**Desmin**	ThermoFisher Rockford, IL, USA	Rabbit	1:100	PA5-16705
**Alexa fluor 488 Anti-rabbit**	Invitrogen Eugene, OR, USA	Goat	1:250 ICC	A32731
**Alexa fluor 488 Anti-mouse**	Invitrogen Eugene, OR, USA	Goat	1:250 ICC	A32731
**Alexa fluor 488 Anti-rabbit**	Invitrogen Eugene, OR, USA	Donkey	1:250 ICC	A10037
**Alexa fluor 647 Anti-goat**	Invitrogen Eugene, OR, USA	Donkey	1:250 ICC	A32814

Table S2

Targeted SNP	Name shRNA	Oligonucleotides for cloning of shRNAs (upper forward and lower reverse oligonucleotide)
**rs1057128**	shA18	ccggaaCACAGGGCCACCTCAACCTtcaagacAGGTTGAGGTGGCCCTGTGtttttttg
		aattcaaaaaaaCACAGGGCCACCTCAACCTgtcttgaAGGTTGAGGTGGCCCTGTGtt
**rs1057128**	shA10	ccggaaGCAGTACTCACAGGGCCACtcaagacGTGGCCCTGTGAGTACTGCtttttttg
		aattcaaaaaaaGCAGTACTCACAGGGCCACgtcttgaGTGGCCCTGTGAGTACTGCtt
**rs1057128**	shA13	ccggaaGTACTCACAGGGCCACCTCtcaagacGAGGTGGCCCTGTGAGTACtttttttg
		aattcaaaaaaaGTACTCACAGGGCCACCTCgtcttgaGAGGTGGCCCTGTGAGTACtt
**rs1057128**	shA15	ccggaaACTCACAGGGCCACCTCAAtcaagacTTGAGGTGGCCCTGTGAGTtttttttg
		aattcaaaaaaaACTCACAGGGCCACCTCAAgtcttgaTTGAGGTGGCCCTGTGAGTtt
**rs1057128**	shA16	ccggaaCTCACAGGGCCACCTCAACtcaagacGTTGAGGTGGCCCTGTGAGtttttttg
		aattcaaaaaaaCTCACAGGGCCACCTCAACgtcttgaGTTGAGGTGGCCCTGTGAGtt
**rs1057128**	shG18	ccggaaCGCAGGGCCACCTCAACCTtcaagacAGGTTGAGGTGGCCCTGCGtttttttg
		aattcaaaaaaaCGCAGGGCCACCTCAACCTgtcttgaAGGTTGAGGTGGCCCTGCGtt
**rs1057128**	shG10	ccggaaGCAGTACTCGCAGGGCCACtcaagacGTGGCCCTGCGAGTACTGCtttttttg

		aattcaaaaaaaGCAGTACTCGCAGGGCCACgtcttgaGTGGCCCTGCGAGTACTGCtt
**rs1057128**	shG11	ccggaaCAGTACTCGCAGGGCCACCtcaagacGGTGGCCCTGCGAGTACTGttttttttg
		aattcaaaaaaaCAGTACTCGCAGGGCCACCgtcttgaGGTGGCCCTGCGAGTACTGtt
**rs1057128**	shG12	ccggaaAGTACTCGCAGGGCCACCTtcaagacAGGTGGCCCTGCGAGTACTttttttttg
		aattcaaaaaaaAGTACTCGCAGGGCCACCTgtcttgaAGGTGGCCCTGCGAGTACTtt
**rs1057128**	shG16	ccggaaCTCGCAGGGCCACCTCAACtcaagacGTTGAGGTGGCCCTGCGAGttttttttg
		aattcaaaaaaaCTCGCAGGGCCACCTCAACgtcttgaGTTGAGGTGGCCCTGCGAGtt
**rs1057128**	shG11m5	ccggaaCAGTACTCGCAGGGGCACCtcaagacGGTGCCCCTGCGAGTACTGttttttttg
		aattcaaaaaaaCAGTACTCGCAGGGGCACCgtcttgaGGTGCCCCTGCGAGTACTGtt
**rs1057128**	shG11m6	ccggaaCAGTACTCGCAGGCCCACCtcaagacGGTGGGCCTGCGAGTACTGttttttttg
		aattcaaaaaaaCAGTACTCGCAGGCCCACCgtcttgaGGTGGGCCTGCGAGTACTGtt
**rs1057128**	shG11m7	ccggaaCAGTACTCGCAGCGCCACCtcaagacGGTGGCGCTGCGAGTACTGttttttttg
		aattcaaaaaaaCAGTACTCGCAGCGCCACCgtcttgaGGTGGCGCTGCGAGTACTGtt
**rs1057128**	shG11fork	ccggaaCAGTACTCGCAGGGCCATAtcaagacGGTGGCCCTGCGAGTACTGttttttttg
		aattcaaaaaaaCAGTACTCGCAGGGCCACCgtcttgaTATGGCCCTGCGAGTACTGtt
**rs1057128**	shG12m5	ccggaaAGTACTCGCAGGGCGACCTtcaagacAGGTCGCCCTGCGAGTACTttttttttg
		aattcaaaaaaaAGTACTCGCAGGGCGACCTgtcttgaAGGTCGCCCTGCGAGTACTtt
**rs1057128**	shG12m6	ccggaaAGTACTCGCAGGGGCACCTtcaagacAGGTGCCCCTGCGAGTACTttttttttg
		aattcaaaaaaaAGTACTCGCAGGGGCACCTgtcttgaAGGTGCCCCTGCGAGTACTtt
**rs1057128**	shG12m7	ccggaaAGTACTCGCAGGCCCACCTtcaagacAGGTGGGCCTGCGAGTACTttttttttg
		aattcaaaaaaaAGTACTCGCAGGCCCACCTgtcttgaAGGTGGGCCTGCGAGTACTtt
**rs1057128**	shG12fork	ccggaaAGTACTCGCAGGGCCACTAtcaagacAGGTGGCCCTGCGAGTACTttttttttg
		aattcaaaaaaaAGTACTCGCAGGGCCACCTgtcttgaTAGTGGCCCTGCGAGTACTtt
**rs1057128**	shG18m10	ccggaaCGCAGGGCCTCCTCAACCTtcaagacAGGTTGAGGAGGCCCTGCGttttttttg
		aattcaaaaaaaCGCAGGGCCTCCTCAACCTgtcttgaAGGTTGAGGAGGCCCTGCGtt

**rs1057128**	shG18m11	ccggaaCGCAGGGCGACCTCAACCTtcaagacAGGTTGAGGTCGCCCTGCGttttttttg
		aattcaaaaaaaCGCAGGGCGACCTCAACCTgtcttgaAGGTTGAGGTCGCCCTGCGtt
**rs1057128**	shG18 fork	ccggaaCGCAGGGCCACCTCAACTAtcaagacAGGTTGAGGTGGCCCTGCGttttttttg
		aattcaaaaaaaCGCAGGGCCACCTCAACCTgtcttgaTAGTTGAGGTGGCCCTGCGtt
**rs1057128**	shA18m11	ccggaaCACAGGGCGACCTCAACCTtcaagacAGGTTGAGGTCGCCCTGTGttttttttg
		aattcaaaaaaaCACAGGGCGACCTCAACCTgtcttgaAGGTTGAGGTCGCCCTGTGtt
**rs1057128**	shA18m10	ccggaaCACAGGGCCTCCTCAACCTtcaagacAGGTTGAGGAGGCCCTGTGttttttttg
		aattcaaaaaaaCACAGGGCCTCCTCAACCTgtcttgaAGGTTGAGGAGGCCCTGTGtt
**rs1057128**	shA18 fork	ccggaaCACAGGGCCACCTCAACTAtcaagacAGGTTGAGGTGGCCCTGTGttttttttg
		aattcaaaaaaaCACAGGGCCACCTCAACCTgtcttgaTAGTTGAGGTGGCCCTGTGtt
**rs8234**	shA8	ccggaaCTGGGCATTACATCGCATAtcaagacTATGCGATGTAATGCCCAGttttttttg
		aattcaaaaaaaCTGGGCATTACATCGCATAgtcttgaTATGCGATGTAATGCCCAGtt
**rs8234**	shA10	ccggaaGGGCATTACATCGCATAGAtcaagacTCTATGCGATGTAATGCCCttttttttg
		aattcaaaaaaaGGGCATTACATCGCATAGAgtcttgaTCTATGCGATGTAATGCCCtt
**rs8234**	shA11	ccggaaGGCATTACATCGCATAGAAtcaagacTTCTATGCGATGTAATGCCttttttttg
		aattcaaaaaaaGGCATTACATCGCATAGAAgtcttgaTTCTATGCGATGTAATGCCtt
**rs8234**	shA12	ccggaaGCATTACATCGCATAGAAAtcaagacTTTCTATGCGATGTAATGCttttttttg
		aattcaaaaaaaGCATTACATCGCATAGAAAgtcttgaTTTCTATGCGATGTAATGCtt
**rs8234**	shA13	ccggaaCATTACATCGCATAGAAATtcaagacATTTCTATGCGATGTAATGttttttttg
		aattcaaaaaaaCATTACATCGCATAGAAATgtcttgaATTTCTATGCGATGTAATGtt
**rs8234**	shA15	ccggaaTTACATCGCATAGAAATCAtcaagacTGATTTCTATGCGATGTAAttttttttg
		aattcaaaaaaaTTACATCGCATAGAAATCAgtcttgaTGATTTCTATGCGATGTAAtt
**rs8234**	shA16	ccggaaTACATCGCATAGAAATCAAtcaagacTTGATTTCTATGCGATGTAttttttttg
		aattcaaaaaaaTACATCGCATAGAAATCAAgtcttgaTTGATTTCTATGCGATGTAtt
**rs8234**	shA18	ccggaaCATCGCATAGAAATCAATAtcaagacTATTGATTTCTATGCGATGttttttttg

		aattcaaaaaaaCATCGCATAGAAATCAATAgtcttgaTATTGATTTCTATGCGATGtt
**rs8234**	shG8	ccggaaCTGGGCATTACGTCGCATAtcaagacTATGCGACGTAATGCCCAGtttttttg
		aattcaaaaaaaCTGGGCATTACGTCGCATAgtcttgaTATGCGACGTAATGCCCAGtt
**rs8234**	shG10	ccggaaGGGCATTACGTCGCATAGAtcaagacTCTATGCGACGTAATGCCCtttttttg
		aattcaaaaaaaGGGCATTACGTCGCATAGAgtcttgaTCTATGCGACGTAATGCCCtt
**rs8234**	shG11	ccggaaGGCATTACGTCGCATAGAAtcaagacTTCTATGCGACGTAATGCCtttttttg
		aattcaaaaaaaGGCATTACGTCGCATAGAAgtcttgaTTCTATGCGACGTAATGCCtt
**rs8234**	shG12	ccggaaGCATTACGTCGCATAGAAAtcaagacTTTCTATGCGACGTAATGCtttttttg
		aattcaaaaaaaGCATTACGTCGCATAGAAAgtcttgaTTTCTATGCGACGTAATGCtt
**rs8234**	shG13	ccggaaCATTACGTCGCATAGAAATtcaagacATTTCTATGCGACGTAATGtttttttg
		aattcaaaaaaaCATTACGTCGCATAGAAATgtcttgaATTTCTATGCGACGTAATGtt
**rs8234**	shG16	ccggaaTACGTCGCATAGAAATCAAtcaagacTTGATTTCTATGCGACGTAtttttttg
		aattcaaaaaaaTACGTCGCATAGAAATCAAgtcttgaTTGATTTCTATGCGACGTAtt
**rs8234**	shG18	ccggaaCGTCGCATAGAAATCAATAtcaagacTATTGATTTCTATGCGACGtttttttg
		aattcaaaaaaaCGTCGCATAGAAATCAATAgtcttgaTATTGATTTCTATGCGACGtt

Table S3

**PCR primers**			
**Gene**	**Forward**	**Reverse**	**Annealing temperature**
***KCNQ1*_wt**	TGAGGATGCTACACGTCGACC	AGCCGATGTACAGGGTGGTTATC	66
***KCNQ1*_mut**	TGAGGATGCTACACGTCGACT	AGCCGATGTACAGGGTGGTTATC	66
***KCNQ1*_total**	TCCTGAGGATGCTACACGTC	AGCCGATGTACAGGGTGGTTATC	66
STAT1	ATCACATTCACATGGGTGGAGC	ACAGATACTTCAGGGGATTCTCAGG	60
OAS1	CATCCGCCTAGTCAAGCACT	AAGACCGTCCGAAATCCCTGG	60

GAPDH	ACCCACTCCTCCACCTTTGAC	ACCCTGTTGCTGTAGCCAAATT	60
HPRT	TGACACTGGCAAAACAATGCA	GGTCCTTTTCACCAGCAAGCT	60
TBP	GCTCACCCACCAACAATTTAG	TCTGCTCTGACTTTAGCACCTG	60
NANOG	AGAATAGCAATGGTGTGACGCAG	TGGATGTTCTGGGTCTGGTTGC	60
OCT4	TGGTTGGAGGGAAGGTGAAG	TGTCTATCTACTGTGTCCCAG	60
OCT4**_viral**	TGTACTCCTCGGTCCCTTTC	CAGGTGGGGTCTTTCATTC	63
SOX2	ACCAATCCCATCCACACTCAC	TCTATACAAGGTCCATTCCCCC	63
SOX2**_viral**	ATCCCAGTGTGGTGGTACG	AAGGCATTCATGGGCCGCTTG	63
MLC2V	ACAACTGACACCAACACCTGC	AGTCCAAGTTGCCAGTCACGTC	63
NKX2.5	TCTATCCACGTGCCTACAGC	AGAAAGTCAGGCTGGCTCAAG	63
KLF4**_viral**	CTGCGGCAAAACCTACACAAA	TTATCGTCGACCACTGTGCTGG	63
MYH6	ACCTGTCCAAGTTCCGCAAGG	TTACAGGTTGGCAAGAGTGAGG	60
MYH7	ACCTGTCCAAGTTCCGCAAGG	TTTGCTGGCACCTCCAGGG	60
